# Supplementary material for: CircADAMTS16 Inhibits Differentiation and Promotes Proliferation of Bovine Adipocytes by Targeting miR-10167-3p
Source: Cells. 2023 Apr 17;12(8):1175. doi: 10.3390/cells12081175 (PMC10136946; doi:10.3390/cells12081175)
Supplement: Supplementary file 1 [file cells-12-01175-s001.zip › cells-2235866 Supplement file 1.pdf]

Chromosome20 Bos-ADAMTS16(XM_024981542) 4891 bp   mRNA CDS: 135..3875

964-1513 >chr20_68316347_68309437_-6910-ADAMTS16

ACATGCCCAAGCCTCCCAAAGAAGACCTTTTCATCCTGCCGGATGAGTATAAATCATGTGTAAGGCAAAAGCGCTCCCTTCTGAAGTCCCAT

AGGAATGAAGAGCTGAATGTGGAGACGCTGGTGGTGGTTGACAAAAAGATGATGCAAAACCATGGCCATGAAAACATCACCACCTACGTGCT

GACAGTTCTCAACATGGTGTCTGCTTTATTCAAAGACGGGACAATAGGAGGGAATATCAACATTGCCATTGTCGCCCTGATTCTTCTGGAAG

AGGAACAGCCAGGGTTGGTGATAAGCCATCACGCGGACCACAGCCTGAGCAGCTTCTGCCAGTGGCAGTCTGGACTGACGGGGAAAGATGGG

GCTCGCCACGACCACGCCATCTTACTCACCGGCCTGGACATATGCTCCTGGAAGAACGAGCCCTGTGACACCCTGGGATTCGCACCCATCAG

TGGCATGTGCAGTAAATACCGCAGCTGTACGATTAATGAGGACACGGGTCTTGGACTGGCCTTCACCATCGCCCACGAGTCTGGACACAA
