# Supplementary material for: CircADAMTS16 Inhibits Differentiation and Promotes Proliferation of Bovine Adipocytes by Targeting miR-10167-3p
Source: Cells. 2023 Apr 17;12(8):1175. doi: 10.3390/cells12081175 (PMC10136946; doi:10.3390/cells12081175)
Supplement: Supplementary file 1 [file cells-12-01175-s001.zip › cells-2235866-supplementary.pdf]

Supplementary Material

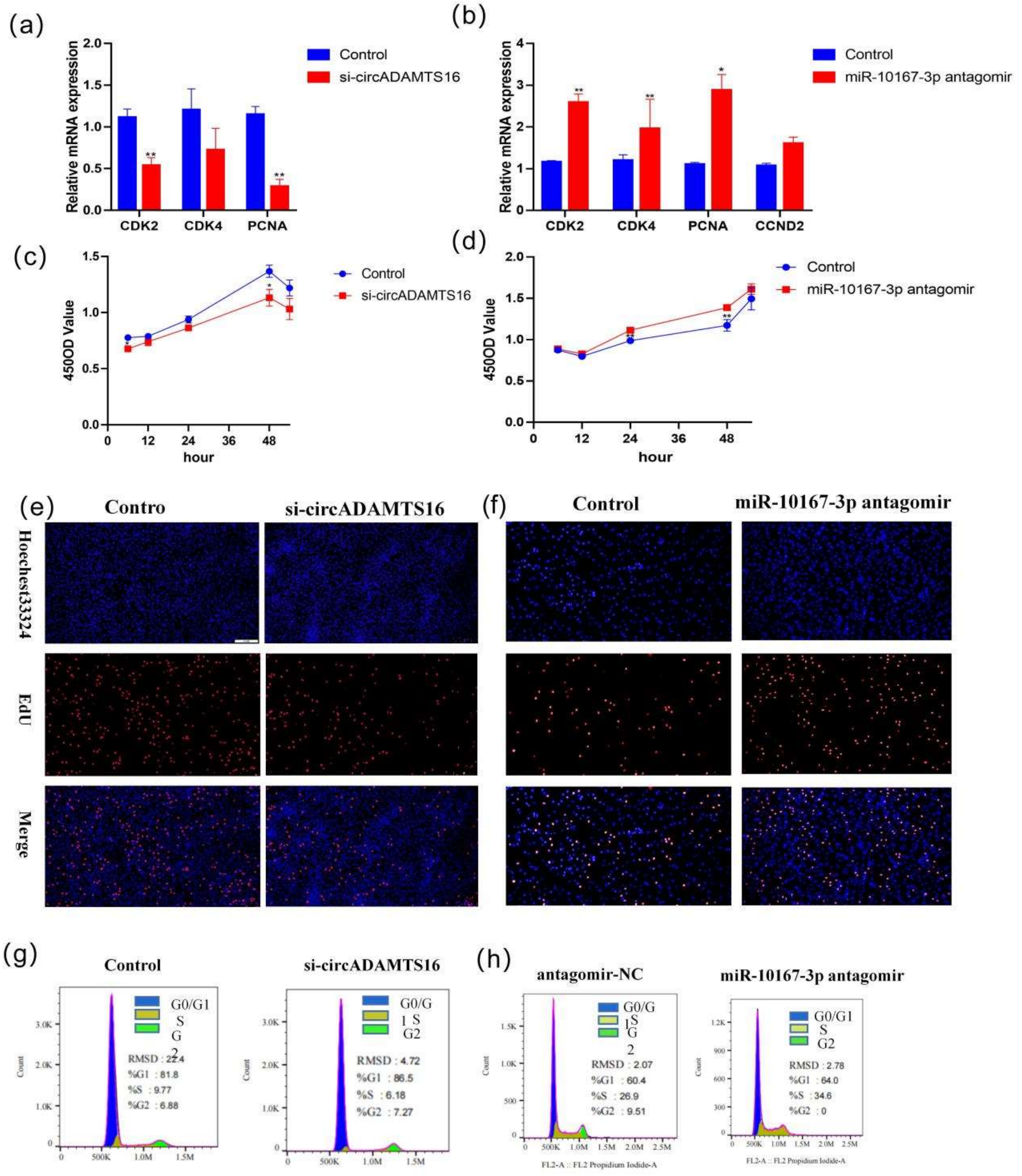

Supplementary figure 1. circADAMTS16 promotes the proliferation of bovine adipocytes by targeting miR-10167-3p. (**a-b**) qPCR was used to detect the mRNA expression levels of proliferation genes *CDK2*, *CDK4*, *PCNA* and *CCND2* after transfection of si-circADAMTS16 and miR-10167-3p antagomir, respectively, (**c-d**) The viability of si-circADAMTS16 and miR-10167 -3p antagomir transfected cells was detected by CCK-8, (**e-f**) si-circADAMTS16 and miR-10167-3p antagomir were transfected into bovine adipocytes respectively, cell proliferation was detected by EdU, (**g-h**) Cell cycle was analyzed by flow cytometry. Scale bar indicates 200 μm. * $P< 0.05$, ** $P<0.01$.

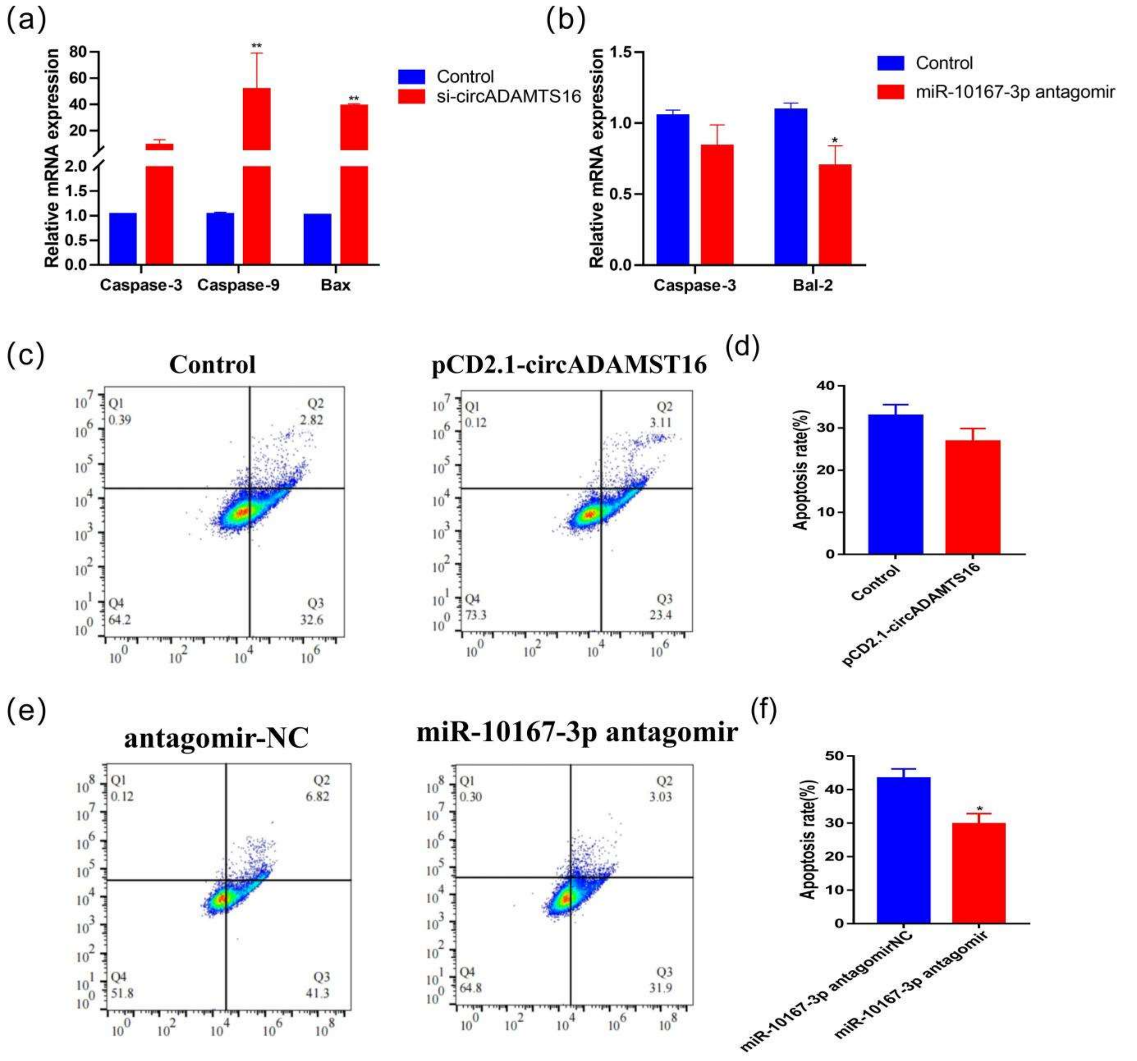

Supplementary figure 2. circADAMTS16 inhibits bovine adipocyte apoptosis by targeting miR-10167-3p. (**a-b**) The mRNA expression levels of apoptotic gene were detected after transfection of si-circADAMTS16 and miR-10167-3p antagomir into bovine adipocytes, respectively, (**c-d**) Apoptosis was assessed by flow cytometry and apoptosis rate following overexpression of circADAMTS16, （**e-f**）Apoptosis assessed by flow cytometry and apoptosis rate after interference with miR-10167-3p. * $P< 0.05$, ** $P < 0.01$.
